# Cyclic Lipodepsipeptides Produced by *Pseudomonas* spp. Naturally Present in Raw Milk Induce Inhibitory Effects on Microbiological Inhibitor Assays for Antibiotic Residue Screening

**DOI:** 10.1371/journal.pone.0098266

**Published:** 2014-05-22

**Authors:** Wim Reybroeck, Matthias De Vleeschouwer, Sophie Marchand, Davy Sinnaeve, Kim Heylen, Jan De Block, Annemieke Madder, José C. Martins, Marc Heyndrickx

**Affiliations:** 1 Institute for Agricultural and Fisheries Research (ILVO), Technology and Food Science Unit, Melle, Belgium; 2 Ghent University (UGent), Department of Organic Chemistry, NMR and Structure Analysis Unit, Gent, Belgium; 3 Ghent University (UGent), Department of Organic Chemistry, Organic and Biomimetic Chemistry Research Unit, Gent, Belgium; 4 Ghent University (UGent), Department of Biochemistry and Microbiology, Laboratory of Microbiology, Gent, Belgium; 5 Ghent University (UGent), Department of Pathology, Bacteriology and Poultry Diseases, Merelbeke, Belgium; Imperial College London, United Kingdom

## Abstract

Two *Pseudomonas* strains, identified as closely related to *Pseudomonas tolaasii*, were isolated from milk of a farm with frequent false-positive Delvotest results for screening putative antibiotic residues in raw milk executed as part of the regulatory quality programme. Growth at 5 to 7°C of these isolates in milk resulted in high lipolysis and the production of bacterial inhibitors. The two main bacterial inhibitors have a molecular weight of 1168.7 and 1140.7 Da respectively, are heat-tolerant and inhibit *Geobacillus stearothermophilus* var. *calidolactis*, the test strain of most of the commercially available microbiological inhibitor tests for screening of antibiotic residues in milk. Furthermore, these bacterial inhibitors show antimicrobial activity against *Staphylococcus aureus*, *Bacillus cereus* and *B. subtilis* and also interfere negatively with yoghurt production. Following their isolation and purification with RP-HPLC, the inhibitors were identified by NMR analysis as cyclic lipodepsipeptides of the viscosin group. Our findings bring to light a new challenge for quality control in the dairy industry. By prolonging the refrigerated storage of raw milk, the keeping quality of milk is influenced by growth and metabolic activities of psychrotrophic bacteria such as pseudomonads. Besides an increased risk of possible spoilage of long shelf-life milk, the production at low temperature of natural bacterial inhibitors may also result in false-positive results for antibiotic residue screening tests based on microbial inhibitor assays thus leading to undue production loss.

## Introduction

Antibiotic residues in milk are of great concern to dairy farmers, milk processors, authorities, and consumers because of public health and industrial implications. In European countries, inhibitory substances are routinely screened in farm milk samples as part of a regulatory quality programme as required in Regulation (EC) No 853/2004 [1] and its Corrigendum [2]. For these purposes, microbiological inhibitory tests are widely used. Their principle is traditionally based on the detection of growth inhibition, indicated by clear inhibition zones in disc assays, or by a colour change of the pH-indicator in the test medium. Many commercial tests like the Delvotest, are based on the last principle and use *Geobacillus stearothermophilus* var. *calidolactis* as test organism and bromocresol purple as pH-indicator. Important drawbacks with microbiological methods are the fact that the identity of the inhibitory substance(s) is not revealed and the possible interference by natural inhibitors.

Inhibitions without any reasonable explanation occur occasionally and can have quite diverse origins, concisely reviewed hereafter. Inhibitory substances other than antibiotics have been reported in milk [3], [4], [5], [6], [7], [8], [9], [10]. Especially colostrum and mastitic milk are known to cause false-positive results in microbiological assays for antibiotic residues [11], [12], [13], [14], [15].

Lactoferrin and lysozyme, two natural antibacterial substances in milk, have been described to separately and synergistically have an inhibitory effect on *Geobacillus stearothermophilus* var. *calidolactis*, the most commonly used test organism in microbiological inhibitor assays [16], [17], [18]. The antibacterial effect of the lactoperoxidase/SCN^-^/H_2_O_2_ system and immunoglobulins is also known [8], [19]. The ability of some vitamin binding proteins to inhibit the growth of bacterial species was suggested by Mullan [20]. Feed complemented with minerals, oligo-elements and vitamins also appear to be a factor that may give rise to false-positive results in the determination of antibiotics in milk with Delvotest SP [21].

Along with the biochemical changes, the physical properties of mastitic milk can also change. Milk with a pH above 7 due to a strong inflammation of the udder and the concomitant transfer of bicarbonate ions from blood to milk [22] can cause false-positive results in microbiological assays based on acid production as criterion for bacterial growth. High concentrations of alkaline disinfecting products or detergents, due to inadequate rinsing and draining, can also induce false-positive screening results [23], [24], [25]. False-positive outcomes can also be induced by high levels of antiparasitic agents or anti-inflammatory products (unpublished data).

Several reports have suggested that the rate of false outcomes increases with increasing somatic cell count (SCC) [26], [27], [28], [29]. Non-proteinaceous inhibitors may be present in early lactation and high SCC milk. In 1963, Stadhouders [30] associated the inhibitory principle in raw milk against starter culture *Streptococcus cremoris* with the fat globules. Due to the activity of indigenous milk lipase or bacterial lipases [31], milk may contain high concentrations of fatty acids, which may inhibit microbial inhibitor tests [32], [33] by their ability to kill or to inhibit the growth of bacteria [34], [35], [31].

*Pseudomonas* spp., which are the most important spoilers of raw milk, can produce heat-stable lipases [36]. A biologically interesting lipid group in milk fat are the polar lipids, which are mainly located in the milk fat globule membrane. In particular, sphingolipids and their derivatives are considered highly bioactive components possessing antibacterial activities [37]. The diapedesis of neutrophils may lead to a leakage of serum components across the mammary epithelium, which, in turn, could lead to lipolysis and inhibitory action [33] and also to an increase in the electrical conductivity of the milk due to an increase in sodium content [27].

Finally, high concentrations of milk protein and milk fat can adversely affect antimicrobial residue test performance to a degree that depends upon the analytical test method used [38]. Higher concentrations of immunoglobulins and milk protein can also cause false-positives with screening tests used on samples from recently freshened heifers or cows [39]. Fat content of milk was positively related to an increase in false-positive rates for the Charm Blue Yellow II, Delvotest Accelerator, Delvotest T, Eclipse 50, and Eclipse 3G [29]. Low protein content may also cause false-positive results, which could possibly be explained by the fact that a minimum protein content is essential for normal growth of the test organism [29].

Recently developed microbiological inhibitor tests show improved detection capabilities for a broad range of antibiotics and chemotherapeutics, allowing improved monitoring of the milk's compliance with regulatory requirements. However, an increased rate of false-positive results with these tests has also been remarked [29]. These can have serious consequences. First, good milk will be discarded since in many cases raw milk is accepted or rejected solely on the basis of a screening test. While subsequent analysis may eventually reveal the causative agent, it cannot influence the decision outcome given the time limitation for storage of raw milk. Second, many countries worldwide will issue a financial penalty to the defaulting farmer as part of the regulatory testing programme, whenever a (false-) positive test result is obtained for inhibitory substances. Recidivism can even result in a temporary interruption of milk collection, leading to production and economic loss. Thus, continued study of agents at the origin of such false positives holds both scientific and economic benefit.

In this work, milk from two farms with frequent problems of false-positive Delvotest MCS results for putative antibiotic residues in the raw milk as part of the regulatory quality programme was thoroughly examined. First analysis showed that no direct link could be established to any of the known interfering parameters described above. Therefore, we aimed to identify the cause of the positive results, so that the farm management could be corrected, the robustness of the microbiological inhibitor test improved and the regulatory testing programme adapted.

## Materials and Methods

### Ethics statement

Since no specific animal experiments were involved in this study, no Institutional Animal Care and Use Committee was approached. For the purpose of this study raw milk was sampled from two Belgian dairy farms. The private farms were owned by Julius De Herdt in Berlaar, Belgium (coordinates: N 51°6′3.505′′, E 4°38′20.617′′) and Christian Simoens in Marquain, Belgium (coordinates: N 50°36′29.332, E 3°19′28.301), respectively. Both farmers, producing milk to be sold to dairies for the production of dairy products, allowed sampling of raw cows' milk from their farm silo for the investigation of the putative presence of residues of antibiotics in the farm milk. At one farm the farmer was helped by ILVO to collect raw milk from eleven individual cows by applying the standard cow milking protocol.

### Milk sampling

Raw milk samples were aseptically collected from the farm cooling tank of two Belgian farms with frequent problems of occurrence of inhibitors in the milk despite no recent use of antimicrobials. On one farm milk from each individual cow (n = 11) was also sampled.

Raw milk, aseptically collected from four individual cows of a farm in the neighbourhood of ILVO, was pooled and used as reference milk ( = blank). The cows in mid-lactation were selected on the basis of not being treated with veterinary drugs during the last months and giving milk with a low number of somatic cells (<2×10^5^ ml^−1^). The blank milk was always checked on the presence of antimicrobials before use with Delvotest MCS or Delvotest SP-NT. In some experiments blank commercial full-cream UHT consumption milk was used.

### Antimicrobial testing by microbiological inhibitor tests and receptor assays

For the detection of residues of antimicrobials in milk, following antibiotic test kits were used: Delvotest SP-NT 5-PACK and Delvotest MCS from DSM-Food Specialties (Delft, the Netherlands); Copan Milk Test microplates from DSM-Food Specialties; PremiTest from DSM-Nutritional Products (Geleen, the Netherlands); Charm MRL Beta-lactam test, Charm II Sulfonamides Milk, Charm II Tetracyclines Milk, Charm II Aminoglycosides Milk, and Charm II Macrolides Milk were from Charm Sciences Inc. (Lawrence, MA). The reagents were stored in a cool room at 4±2°C, except for the microbiological inhibitor tests that were stored between 6 and 15°C.

Incubation of the tests (Plates of Delvotest SP-NT, Delvotest MCS, Copan Milk Test, and ampoules of Premitest) took place in a covered water bath (Type 19+ MP thermostat from Julabo Labor-technic GmbH (Seelbach, Germany)) at 64.0±1°C. *Geobacillus stearothermophilus* disc assays were incubated in an incubator BD240 with natural convection from Binder GmbH (Tittlingen, Germany) at 55.0±1°C.

The colour interpretation of Delvotest SP-NT and Delvotest MCS plates was done by means of a flatbed scanner (HP Scanjet 7400C, Hewlett-Packard Company, Palo Alto, CA) connected to DelvoScan software, version 3.05 (DSM-Food Specialties). The cut-off was set at a Z-value  = −3.00. A yellow colour of the agar after incubation indicates no presence of inhibitory substances (negative) and as a consequence a Z-value <cut-off; when inhibitors are present in the milk the colour of the pH-indicator in the agar remains blue/purple, resulting in Z-values >−3.00. For the reading of Copan Milk Test plates a HP GRLYB-0307 flatbed scanner (Hewlett-Packard Company) connected to CScan software, version 1.32 (Copan Italia S.p.A.) was used. The cut-off was set at a CIF (Colour Impact Factor; yellow  = 0.1 and purple  = 10)-value  = 4.5. The colour of PremiTest ampoules was interpreted by means of a flatbed scanner (HP Scanjet 7400C, Hewlett-Packard Company) connected to PremiScan software, version 1.02 (DSM-Premitest B.V., Heerlen, the Netherlands). The cut-off was set at a Z-value  = −4.50.

Reagents of Charm MRL Beta-lactam test were incubated in a ROSA Incubator at 56°C (Charm Sciences Inc.). The strips were interpreted by means of a ROSA Reader (Charm Sciences Inc.) with a reader value  = 0 as cut-off. Charm II reagents were incubated in a Charm Inctronic 2 Dual Incubator (Charm Sciences Inc.). The amount of bound radioactive tracer was read with a liquid scintillation counter 1409 (Wallac, Waltham, MA). The cut-off cpm for each type of test was set according to the protocols provided by the reagents manufacturer.

All commercial microbial inhibitor tests were used following the instructions of the kit manufacturers. In every run of each inhibitor test, blank reference milk and antibiotic standards were included. The *p*-aminobenzoic acid (A9878) was from Sigma-Aldrich (Bornem, Belgium); the penicillinase (L037) was from Genzyme (West Malling, UK). The disc assay was prepared and performed as described by Ginn *et al*. [40]. For the disc assay an inhibition zone around the filter disc of ≥2.0 mm is considered as positive.

### Milk quality assessment

#### Determination of milk composition, somatic cell count, and pH

The fat, protein and lactose content of the milk samples was determined by Milcoscan 4000 (FOSS, Hillerød, Denmark) and the somatic cell count (SCC) by Fossomatic 5000 (FOSS) at vzw Melkcontrolecentrum-Vlaanderen (Lier, Belgium). A SevenMulti pH meter was used from Mettler-Toledo Inc. (Columbus, OH) for pH-measurements. In Europe, the norm for somatic cells is ≤4×10^5^ ml^−1^; the criterion is applied on rolling geometric averages (Corrigendum to Regulation (EC) No 853/2004 [2]).

#### Total bacterial count, enumeration of psychrotrophic and lipolytic bacteria

The total bacterial count and enumeration of the psychrotrophic and fat splitting bacteria were performed on Plate Count Agar (PCA, 3d at 30°C), Violet Red Bile Agar (VRBA, 1d at 30°C), PCA (7d at 7°C), and Tributyrin Agar (TBA, 3d at 30°C), respectively, following ISO 4833 [41], ISO 4832 [42], ISO 6730/IDF101 [43], and Method 3.32 [44], respectively. PCA (CM0325), VRBA (CM0107), and TBA (PM0004) were from Oxoid Limited (Basingstoke, UK). In Europe, the norm for raw cows' milk for total bacterial count is ≤10^5^ cfu ml^−1^; the criterion is applied on rolling geometric averages (Corrigendum to Regulation (EC) No 853/2004 [2]).

#### Determination of lipolysis, fat oxidation and proteolysis

The lipolysis in milk was estimated as described by Driessen *et al*. [45]. Therefore the milk fat was extracted according to IDF Bulletin 265 [46] using the Bureau of Dairy Industries (BDI)-reagent. Afterwards the free fatty acids were titrated with NaOH and phenolphthalein as indicator according to IDF Standard 6B [47]. The free fatty acids content is expressed as mass% oleic acid 100 g^−1^ fat. The peroxide value was determined following AOAC Official Method 965.93 [48] and expressed in meq O_2_ kg^−1^ fat. The proteolysis in milk was monitored following an adaptation of the method described by Polychroniadou [49].

#### Isolation, characterization and identifications of bacterial isolates

Two strains, *Pseudomonas* P866 and P867, were isolated from the VRBA plates for the enumeration of the number of coliforms in some raw milk samples. These strains were purified, identified and conserved at −80°C in Protect vials (International Medical Products, Brussels, Belgium).

Single colonies were streaked out on different selective media to visualise enzymatic activities: Tributyrin Agar for lipolysis [44] and PCA +2% UHT milk for the detection of proteolysis (FNZ Method 53.27 [50]). The hemolytic activity was determined on Blood Agar (BA) plates with addition of 5% of sheep blood as described in ISO Standard 7932 [51]. Display of a clear halo around the colony was considered as positive. Blood Agar Base No 2 (BA, CM0271) and Sheep blood (SR0051) were from Oxoid Limited. The ability to use lactose was checked by the isopropyl-β-D-thio-galactoside-test (IPTG) [52].

Identification of the strains was done with API 20NE (20050) from bioMérieux France (Craponne, France) and *rpoB* sequencing. The *rpoB* gene was amplified as described previously by Ait Tayeb *et al*. [53]. The PCR-amplified *rpoB* gene products were purified using the Nucleofast 96 PCR system (Millipore, Billerica, MA). For each sequence reaction a mixture was made using 3 µl purified and concentrated PCR product, 1 µl of BigDye Termination RR mix version 3.1 (Applied Biosystems, Carlsbad, CA), 1.5 µl of BigDye buffer (5×), 1.5 µl sterile milliQ water, and 3 µl (20 ng µl^−1^) of one of the sequencing primers. The amplification primers were used as sequencing primers. The temperature-time profile was as follows: 90 s at 94°C; 30 cycles of denaturation for 10 s at 94°C, primer annealing for 20 s at 45°C and extension for 50 s at 72°C; and a final extension of 5 minutes at 72°C. The sequencing products were cleaned up as described previously [54]. Sequence analysis of the *rpoB* gene was performed using a 3100 DNA Sequencer (Applied Biosystems) according to protocols provided by the manufacturer.

#### Phylogenetic analysis

Forward and reverse strands of *rpoB* were assembled with the BioNumerics 4.6 software (Applied Maths, Sint-Martens-Latem, Belgium) and were aligned with sequences retrieved from the EMBL database using ClustalX [55]. Phylogenetic analyses were performed with Treecon [56]. Trees were constructed with the neighbour joining algorithm without corrections. Statistical evaluation of the tree topologies was performed by bootstrap analysis with 1000 resamplings.

#### Growth and bacterial inhibitor production

Growth and bacterial inhibitor production was followed in raw milk, full-cream UHT milk, and Brain Heart Infusion Broth (BHI, CM1135, Oxoid Limited). After inoculation with *Pseudomonas* P866 or P867, the medium was incubated at 5–7 and 30°C with daily sampling for enumeration of total bacterial count while the bacterial inhibitor production was followed by Delvotest SP-NT, PremiTest, or on the disc assay with *Geobacillus stearothermophilius* var. *calidolactis*. Bacterial inhibitor production of *P. tolaasii* LMG 2342^T^ in full-cream UHT milk was checked on Delvotest.

The production of bacterial inhibitor was also followed for pure cultures of *Pseudomonas* P866 and P867, for P866 and P867 in competition with raw milk flora, and for P867 in competition with a mixture of 10 psychrotrophic *Pseudomonas* strains isolated from Belgian raw milk samples.

### Bacterial inhibitor characterization assays

#### Estimation of the molecular weight

The molecular weight of the bacterial inhibitor was tested by means of regenerated cellulose dialysis membrane with a molecular weight cut-off (MWCO) of 1 kDa (Spectra/Por Dialysis membrane 6 (132638) from Spectrum Laboratories, Inc. (Rancho Dominguez, CA)). Full-cream UHT milk was inoculated with *Pseudomonas* strain P867 and incubated for 24 h at 30°C. Dialysis of positive UHT milk against blank raw milk was performed. Milk from the in- and outside the dialysis membrane was sampled after 24 hours at 4°C to be tested on Delvotest SP-NT.

#### Heat tolerance of bacterial inhibitor and fatty acids

The heat tolerance of the bacterial inhibitor produced by *Pseudomonas* P866 and P867 was tested by heating 5 ml of the skimmed fraction of full-cream UHT milk after growth of the bacteria in a glass test tube in a water bath at different temperatures. After heating at 80, 90, or 100°C for 10 min, the milk samples were rapidly cooled to 20°C with cold water and tested on Delvotest SP-NT.

The interference and heat tolerance of free fatty acids on the disk assay, Delvotest SP-NT and Copan Milk Test was tested by spiking blank raw milk with 0.15% (w/v) of the following fatty acids from Sigma-Aldrich: myristic (M3128), oleic acid (O1008), palmitic (P0500), and stearic acid (S4751). Part of the doped milk was preheated before testing.

#### Inhibition spectrum of the bacterial inhibitor

For applications, not directly related to the disturbance of microbiological inhibitor tests, the antimicrobial activity was tested against following bacterial *species*: *Bacillus cereus* LMG 8221, *Bacillus subtilis* LMG 7135^T^, *Escherichia coli* LMG 2092^T^, *Geobacillus stearothermophilus* var. *calidolactis* LMG 11163, *Listeria monocytogenes* ATTC 19110^T^, *Pseudomonas fluorescens* LMG 1794^T^, *Salmonella* Enteritidis LMG 10396^T^, *and Staphylococcus aureus* LMG 8074.

BHI broth was inoculated with *Pseudomonas* strain P867, incubated for 24 h at 30°C and filtered through a Millipak 0.22 µm Filter Unit (Millipore corporation). The inhibition spectrum of the bacterial inhibitor produced was tested by placing 12.7 mm diameter filter discs (Antibiotic Test Discs, FN0905A00005, Novolab, Geraardsbergen, Belgium) impregnated with 80 µl of filtrate on PCA plates, filled with 10 ml of agar and inseminated with different bacterial strains. The plates were incubated for 24 hours at 30°C, except for the plates with *Geobacillus stearothermophilus* var. *calidolactis*, which were incubated for 24 hours at 55°C.

### Bacterial inhibitor isolation

For easy purification of the inhibitor substance, the isolated strain was inoculated in Maximum Recovery Diluent (CM0733, Oxoid Limited) and incubated for 3–4 days at 30°C, to ensure maximal inhibitor production. The bacterial cells were removed by centrifugation (20 minutes at 5,500 g).

For NMR identification of the inhibitor, *Pseudomonas* strain P867 was grown on *Pseudomonas* agar (PSA) plates (Oxoid, CM0559 + CFC Selective Agar Supplement (Oxoid, SR103)) for 48 h at 30°C for 48 h at 30°C. Cell material was collected in sterile distilled water and pelleted by centrifugation (20 minutes at 5,500 g). Part of the supernatans was, after addition of inhibitor-free milk powder, tested on inhibitory characteristics on Delvotest T. The remaining part of the cell-free supernatant was acidified; the precipitate was obtained by centrifugation and twice washed as described by Tran *et al.* [57]. The crude peptide mixture was further purified by preparative RP-HPLC. Samples were dissolved in Milli-Q water (Millipore Corporation) with pH adjusted to 8.0 with NaOH, until a concentration of 50 mg/ml was obtained. Eight injections of 1 ml were performed on a Phenomenex column (Luna C18(2) 250×21.2 mm, 5 µm particle size) operating at a flow rate of 17.5 ml/min. The ratio of the mobile phase solvents, H_2_O (5 mM NH_4_OAc)/CH_3_CN was changed in a linear fashion from 25∶75 to 0∶100 over a time span of 15 minutes. The major compounds (**1** and **4**) were collected in two fractions (A and C) following UV detection at λ = 214 nm. Some additional co-eluting minor compounds (**2** and **3**) were collected in a third fraction (B).

### Bacterial inhibitor characterization

Initial LC-MS data following extraction but prior to purification, were collected with an Agilent 1100 Series HPLC with an ESI detector type VL, equipped with a Phenomenex Inc. (Torrance, CA) column (Luna C18(2), 250×4.60 mm, 5 µm particle size) The flow rate was 1 ml/min. The mobile phase solvents, 5 mM ammonium acetate in water and acetonitrile, were linearly changed from a 25∶75 ratio to a 0∶100 ratio over a time span of 15 minutes. High-resolution mass spectra were recorded on an Agilent 6220A time-of-flight mass spectrometer (Agilent, Santa Clara, CA), equipped with an Agilent ESI/APCI multimode source. The ionization mode was set to APCI (atmospheric pressure chemical ionization), while the mass spectra were acquired in 4 GHz high-resolution mode with a mass range set to 3200 Da. NMR measurements on lipopeptide fractions A, B and C were performed on respectively samples of 3.3 mg, 3.6 mg and 5.7 mg dissolved in 600 µl DMF-d7 (Eurisotop, Saint Aubin, France). NMR experiments were performed on either a Bruker Avance II spectrometer (Bruker Biospin, Billerica, MA) operating at 700.13 MHz and 176.05 MHz for ^1^H and ^13^C respectively and equipped with a 5 mm ^1^H,^13^C,^15^N TXI-Z probe. Additional measurements in acetone-d6 were executed to enable chemical shift comparison with literature data of massetolide compounds. These were performed on a Bruker Avance III spectrometer operating at 500.13 MHz and 125.76 MHz for ^1^H and ^13^C respectively, and equipped with a 5 mm ^1^HBBI-Z probe. The sample temperature was set to 25°C throughout. 2D spectra measured for structure elucidation include a ^1^H–^1^H gCOSY, ^1^H–^1^H TOCSY with a 90 ms MLEV–17 spinlock, a sensitivity-improved, multiplicity edited, ^1^H–^13^C gHSQC applying adiabatic 180° pulses, a ^1^H–^1^H off-resonance ROESY with a 300 ms spinlock and a ^1^H–^13^C gHMBC experiment optimized for a ^n^J_CH_ coupling of 7 Hz. Standard pulse sequences as present in the Bruker library were used throughout, except for the ROESY where an in-house sequence was used. Typically, 2048 data points were sampled in the direct dimension for 512 data points in the indirect one, with the spectral width respectively set to 11 ppm along the ^1^H dimension and 110 ppm (gHSQC) or 220 ppm (gHMBC) along the ^13^C dimension. For 2D processing, the spectra were zero filled to obtain a 2048×2048 real data matrix. Before Fourier transformation, all spectra were multiplied with a squared cosine bell function in both dimensions, except for the gCOSY and gHMBC spectra where a squared sine bell was applied together with magnitude calculation.

#### Inhibition by purified lipodepsipeptide fractions

Small quantities of compounds isolated from fractions A, B and C were freeze-dried and redissolved in a small quantity of raw milk and tested (5 replicates) on Delvotest SP-NT to establish whether the inhibitory characteristics are linked to the purified lipopeptides **1–4**.

## Results

### Screening of milk for antibiotic residues

Milks sampled on two farms with frequent penalizations for inhibitory substances in the farm silo milk tested (low) positive (presence of inhibitory substances) on Delvotest MCS, but negative (absence of inhibitory substances) on Copan Milk Test, but with higher CIF values than for blank milk samples. Even after pre-heating the milk at 80°C for 10 min, removal of the fat after centrifugation or addition of penicillinase or *p*-aminobenzoic acid to the milk, still positive Delvotest MCS results were obtained. The milk samples were further tested with Charm MRL Beta-lactam test and Charm II assay for sulfonamides, tetracyclines, aminoglycosides, and macrolides in milk, all with negative results. The presence of antibiotic residues of the most important antibiotic families could not be established.

The results of the second sampling of milk from each individual cow (n = 11) and the farm silo (in duplicate) at farm 1 are presented in Table 1. All individual cow milk samples tested negatively on Delvotest MCS the day after sampling except for cow 3, giving a borderline positive Z-value (−0.24). This positive result could be explained by the higher pH of the milk (7.06), characteristic for subclinical mastitis, since the growth of *Geobacillus* in the Delvotest is followed with bromocresol purple, a pH indicator. The farm silo milk, sampled in duplicate, tested negative that day, but the Z-values (−3.32 and 3.47) were close to the cut-off of −3.00 indicating some inhibition of the Delvotest test organism *Geobacillus stearothermophilus* var. *calidolactis*. Retesting the farm silo and individual cow milk samples, stored at 4°C, three days after sampling, resulted in positive Delvotest MCS results for the farm silo milk samples and the milk of cow 3 and 9. For the milk of cows 4 and 7, borderline negative results were obtained. All milk samples tested negative on Copan Milk Test (data not shown), a more robust microbiological inhibitor test.

**Table 1:** Quality parameters (total bacterial count, somatic cell count, and pH) and Delvotest MCS (SP-NT) results (1 and 3 days after sampling), of reference blank milk, of 11 individual cow milk samples, and of 2 samples of the farm silo milk, from a Flemish farm frequently penalized for antimicrobials in the farm silo milk.

Milk	Total	Somatic	pH	Delvotest MCS		Delvotest MCS	
	bacterial	cell		(d+1)^a^		(d+3)^a^	
	count	count		(Z-value)		(Z-value)	
	(cfu ml^−1^)	(×10^3^ ml^−1^)		mean	SD	mean	SD
cow 1	1,100	409	6.71	−7.90	0.12	−4.14	0.08
cow 2	350	20	6.52	−9.66	0.18	−6.18	0.16
cow 3	8,300	959	7.06	−0.24^b^	0.13	2.61^b^	0.61
cow 4	40	314	6.86	−5.90	0.45	−3.87	2.25
cow 5	16,000	251	6.70	−5.15	0.39	−4.08	1.69
cow 6	4,900	12	6.60	−7.98	0.23	−6.39	0.13
cow 7	6,200	626	6.86	−7.34	0.45	−3.77	0.12
cow 8	4,900	1,304	6.81	−7.22	0.08	−4.61	0.32
cow 9	7,900	1,407	6.76	−7.56	0.06	−2.67^b^	0.49
cow 10	70	36	6.62	−7.59	0.09	−4.67	0.04
cow 11	6,800	643	6.69	−6.97	0.00	−4.75	0.21
silo	1,510	168	6.64	−3.32	0.09	−0.10^b^	0.33
silo	1,290	179	6.65	−3.47	0.49	0.21^b^	0.32
blank milk	na^c^	159	6.80	−8.96	0.52	−6.87	0.31

Delvotest cut-off Z-value = −3.00 Delvotest results based on two replicates, except for blank milk (3 replicates).

Notes: ^a^ d+, days after sampling; ^b^, positive result; ^c^ na, no data available

### Assessment of milk quality

The farm silo milk samples were also analysed on composition and quality. The somatic cell counts, 300,000 and 273,000 ml^−1^, respectively, were below the norm of 400,000 ml^−1^ but above the country average of 190,000 ml^−1^. At 6.4 and 6.6, the pH values were slightly lower than the average value for raw milk. The content of free fatty acids was 1.8 and 3% of the fat (≈0.15% of the milk), respectively. These free fatty acid concentrations were high for raw milk and may be indicative for the activity of bacterial lipases. Both silo milk samples showed a normal proteolysis, and a normal fat oxidation.

Analytical results of the second sampling of milk from each individual cow (n = 11) and the farm silo (in duplicate) at farm 1 are presented in Table 1. High SCC above the norm of 4×10^5^ ml^−1^ were measured in 6 out 11 individual cow milk samples. A high level of free fatty acids was present in the milk of cow 9 and in both farm silo milk samples.

Regarding the microbiological quality of the individual cow milk samples, no extreme values were found for the total bacterial count (40–16,000 cfu ml^−1^), the number of coliforms (<1–29 cfu ml^−1^, data not shown), psychrotrophic (<10–2,000 cfu ml^−1^, data not shown), and fat splitting bacteria (<10–4,100 cfu ml^−1^, data not shown). The composition parameters fat, protein, and lactose (data not shown) of the silo milk were normal.

### Isolation, characterization and identification of strains

**Figure 1:**
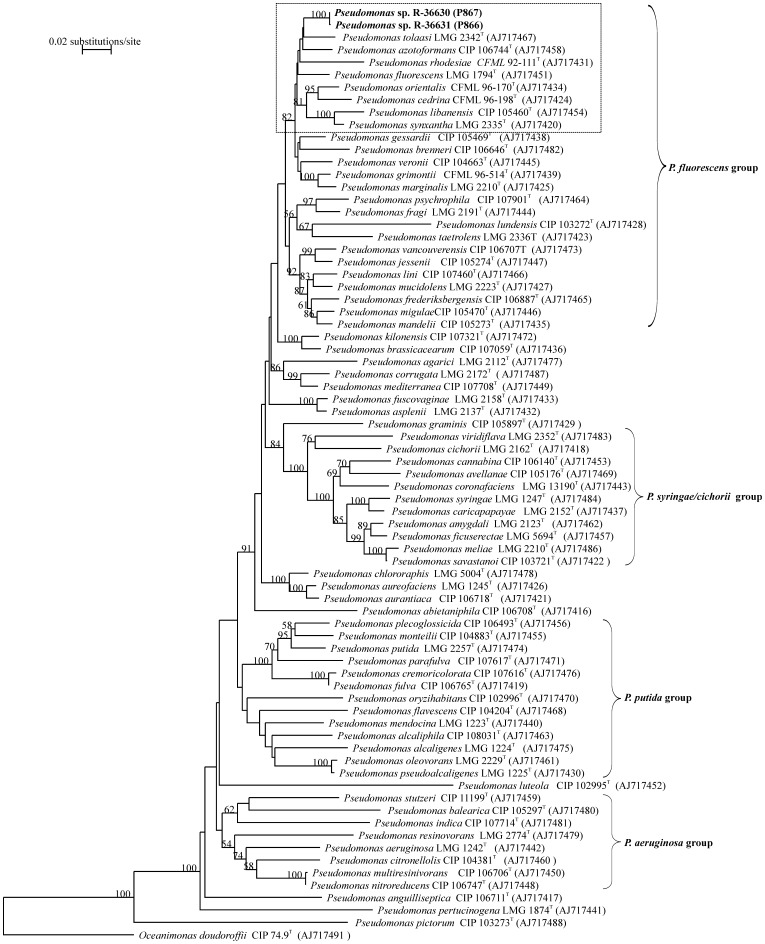
Phylogenetic analysis of the *Pseudomonas* isolates P866 and P867 on the basis of *rpoB* sequences. Unrooted neighbour joining tree was based on partial *rpoB* sequences (1030 bp). Bootstrap values were generated from 1000 replicates of neighbour joining. Bootstrap values higher than 50% are given.

On VRBA plates, coliforms characteristically form dark red colonies usually surrounded by a reddish zone with a granular appearance by precipitation of bile salts. However, on some plates some bacteria gave atypical results with a clear halo between the colony and the reddish zone. P866 and P867 (*rpoB* sequence accession numbers R-36630 and R-36631, respectively), isolated as atypical bacterial strains from the VRBA plates with milk of cow 3 and 4 were purified and identified as *Pseudomonas fluorescens* with a similarity level of 99.9% by using API 20NE. Since API identification is limited in comparison to sequence-based identification [58], *rpoB* sequence analysis was performed. A phylogenetic clustering with all public available *rpoB* sequences from members of the *Pseudomonas* genus indicated *P. tolaasii* LMG 2342^T^ as the closest relative for both strains (Figure 1).

The strains were able to grow at 7°C (psychrophilic) and did not show any lipase activity using tributyrin as substrate. Both strains showed a very strong hemolytic activity on blood agar plates; also a proteolytic activity was detected. The *P. tolaasii* LMG 2342^T^ strain did not show any hemolytic activity, a finding in line with the observations of Munsch and Alatossava [59] whom also reported a non-haemolytic activity for a non-pathogenic variant of *P. tolaasii* LMG 2342^T^.

None of the bacteria were able to hydrolyse lactose. The pH of raw milk inoculated with *Pseudomonas* P867 dropped from 6.70 to 6.53 after 48 hours incubation at 30°C. Also fat oxidation was observed in the inoculated milk incubated for 1 day at 30°C or for 4 days at 5°C (inoculated milk: 0.46 (at 30°C) and 1.08 (at 5°C) meq O_2_ kg^−1^ fat; reference milk: 0.15 meq O_2_ kg^−1^ fat).

### Growth and bacterial inhibitor production

The growth and inhibitor production of *Pseudomonas* strains P866 and P867 inoculated in full-cream UHT milk were monitored at 5°C, 7°C and also at 30°C. As could be expected, both parameters were strongly influenced by the number of bacteria in the inoculum. Even so, for a similar number of bacteria inoculated, the growth and the bacterial inhibitor production were not constant. An example for the growth of *Pseudomonas* strain P867 incubated at 7°C is shown in Table 2.

**Table 2:** Growth and bacterial inhibitor production by *Pseudomonas* strain P867 in full-cream UHT milk at 7°C.

Time	Bacterial	Delvotest SP-NT		
(days)	count	(Z-value)		result
	(cfu ml^−1^)	mean	SD	
t = 0	7.0×10^2^	−10.24	0.30	neg
t = 1	5.6×10^5^	−10.10	0.14	neg
t = 2	1.6×10^7^	−10.32	0.05	neg
t = 3	4.5×10^7^	−9.19	0.01	neg
t = 4	1.3×10^8^	−4.93	0.54	neg
t = 5	6.0×10^7^	2.18	1.07	pos

Delvotest cut-off Z-value = −3.00. Delvotest results based on two replicates.

Notes: pos, positive; neg, negative.

Milk inoculated with P866 or P867 and incubated at 7°C, needed 5 days and a high bacterial count to become positive on Delvotest. Also in growth experiments at 30°C, a high number of bacteria (>10^7^ ml^−1^) were needed before positive Delvotest results could be generated (data not shown).

In most growth experiments the highest production of bacterial inhibitor production was obtained in the stationary growth phase.

The *Pseudomonas* strains P866 and P867 do not need milk for bacterial inhibitor production. A culture of *Pseudomonas* P866 and P867 grown in BHI broth tested positively on PremiTest, a tissue test comparable to the Delvotest.

### Bacterial inhibitor characterization assays

In the dialysis experiments, milk with bacterial inhibitors by growth of *Pseudomonas* strain P867 (Z-value  = 10.77) inside the dialysis membrane became negative on Delvotest SP-NT (Z-value  = −5.60) after 24 hours dialysis at 4°C against blank raw milk; while in the inverse dialysis blank raw milk (Z-value  = −6.68) became positive on Delvotest SP-NT (Z-value  = 8.08). The results of the dialysis experiments show that the bacterial inhibitor is able to permeate the 1 kDa dialysis membrane.

Heating of milk (5 min at 82°C or 10 min at 80°C) inactivates natural inhibitors and is often applied to prove false-positive results in microbial growth inhibition assays [60]. The bacterial inhibitors produced by *Pseudomonas* P866 and P867 showed to be heat-stable at 100°C for 10 min.

On *Geobacillus* disc assay plates, the culture of *Pseudomonas* strain P867 in milk caused inhibition of the Gram-positive *Bacillus cereus* LMG 8221, *Geobacillus stearothermophilus* var. *calidolactis* LMG 11163, *Bacillus subtilis* LMG 7135^T^, and *Staphylococcus aureus* LMG 8074. No inhibition zones were found on the plates inseminated with *Listeria monocytogenes* ATTC 19110^T^ (Gram-positive) or the Gram-negative *Escherichia coli* LMG 2092^T^, *Pseudomonas fluorescens* LMG 1794^T^, or *Salmonella* Enteritidis LMG 10396^T^.

### Isolation and identification of the bacterial inhibitor

LC-MS analysis of semi-purified extracts revealed the presence of at least four compounds that could be separated in three fractions: fraction A (compound **1**, 1140.7 Da), fraction B (two co-eluting minor compounds **2** and **3**, 1154.7 Da each) and fraction C (compound **4**, 1168.7 Da) (Figures S1 and S2 in File S1). Structure elucidation by NMR identified these compounds as four similar cyclic lipodepsipeptides (CLPs), as discussed here in detail for the fourth, most abundant compound.

**Figure 2:**
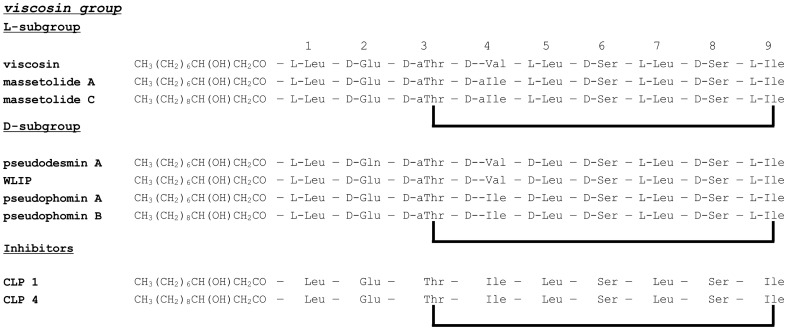
Structures of a selected number of known viscosin group CLPs and the obtained inhibitors with established molecular constitution.

The NMR 1D ^1^H spectrum (Figure S3 in File S1) and resonance assignment (Table S1 in File S1) in DMF-d7 are provided in the supporting information. Analysis of the characteristic amino acid correlation patterns observed in the 2D ^1^H–^1^H TOCSY spectrum of **4** (Figure S4 in File S1) revealed the presence of three Leu, two Ile, two Ser, one Thr and one Glu. The configuration is not further specified since the applied NMR techniques only reveal the amino acid constitution. This is of relevance since CLPs typically contain both L- and D-amino acids, with variations in this respect between similar analogues (Figure 2). Combined with the ^1^H–^13^C gHSQC spectrum, the presence of a spin system corresponding to a linear 3-hydroxy fatty acid (FA) moiety was also revealed. By following the sequential H^α^-H^N^ cross-peaks in the 2D ^1^H–^1^H off-resonance ROESY spectrum (Figure S5 in File S1), the N→C amino acid sequence and the FA attachment could be established as FA-Leu1-Glu2-Thr3-Ile4-Leu5-Ser6-Leu7-Ser8-Ile9. The ^1^H–^13^C gHMBC spectrum subsequently allowed the assignment of most carbonyl ^13^C resonances from the ^2^J_CH_ coupling with the H^N^ resonances. Furthermore, the existence of a correlation between the C-terminal Ile9 carbonyl ^13^C and the Thr3 H^β^ resonances in the HMBC spectrum and the unusually high chemical shift value of the Thr3 H^β^ resonance [61] indicates the presence of an ester bond between the C-terminal carboxylic acid and the Thr3 hydroxyl groups (Figures S5 and S6 in File S1). Due to significant overlap of methylene groups in the ^1^H-^13^C HSQC, the length of the fatty acid chain could only be deduced from the unaccounted part of the chemical formula C_56_H_99_N_9_O_16_ obtained from HR-MS (Figure S7 in File S1). By comparison with the known structural features at this point, the fatty acid is identified as a 3-hydroxydodecanoic acid moiety (HDDA), finalizing the constitutional structure elucidation.

Structural analysis of **1** was achieved in an analogous fashion (Figures S8 and S9, Table S2 in File S1) and was found to differ from **4** by the presence of 3-hydroxydecanoic acid (HDA) instead of a HDDA moiety. The reduction in chain length with two methylene units explains the 28 Da mass reduction compared to **4**. As the minor CLPs **2** and **3** are co-eluting, NMR spectra could only be measured on an approximately equimolar mixture. The resonances of both compounds were insufficiently resolved to unambiguously assign these to one particular peptide. However, most of the ^1^H and ^13^C chemical shifts of both compounds were practically identical to those of **4**, while HR-MS data revealed their chemical formula to be equal and 14 Da less compared to **4** (Figure S10 in File S1). Their characterisation was achieved through direct comparison with the 2D spectra of **4**. This reveals one of the two compounds to possess an Ile4→Val4 substitution and one an Ile9→Val9 substitution. Since the HR-MS data suggests a loss of 14 Da for both **2** and **3** relative to **4**, it appears reasonable to assume the presence of a single Ile/Val substitution in both **2** and **3**. Thus, the sequences of **2** and **3** are most likely C_12_-Leu1-Glu2-Thr3-Ile4-Leu5-Ser6-Leu7-Ser8-Val9 and C_12_-Leu1-Glu2-Thr3-Val4-Leu5-Ser6-Leu7-Ser8-Ile9. Since spectral complexity precludes extraction of the fatty acid chain length from the NMR spectra, the alternative sequences C_13_-Leu1-Glu2-Thr3-Val4-Leu5-Ser6-Leu7-Ser8-Val9 and C_11_-Leu1-Glu2-Thr3-Ile9-Leu5-Ser6-Leu7-Ser8-Ile9 can presently not be excluded.

Finally, the purified compounds **1**, **2**+**3** and **4** issued from fractions A, B and C respectively, were reconstituted in blank raw milk and tested on Delvotest SP-NT. The Z-values obtained were: blank milk: -11.55±0.64; fraction A: -0.58±0.64; fraction B: -1.88±0.35 and fraction C: 1.99±1.23 proving that the inhibitory characteristics of each fraction can be lined to the purified lipodepsipeptides.

## Discussion

Microbiological inhibitor tests can generate false-positive results. In the European residue legislation no maximum norm is fixed for screening tests for the rate of false non-compliant results. Only requirements for the rate of false compliant results are stipulated. Following Commission Decision 2002/657/EC [62] this rate should be <5% (β-error) at the level of interest. In the same Commission Decision, a general requirement for specificity states that any valid method should be able to distinguish between the analyte (antibiotic residue) and the other substances under the experimental conditions used and that it is of prime importance that interference, which might arise from matrix components, is investigated. Finally, the same Commission Decision states that in case a suspected non-compliant screening result is obtained, this result should be confirmed by a confirmatory method. In practice however, this is not always achievable, especially since raw milk cannot be maintained in quarantine for the required time. Therefore a decision about the fate of raw milk is generally based on a screening test result.

Farm tank milk from two Belgian dairy producers, penalized on several occasions because their milk tested positive for the presence of antimicrobials in the Delvotest MCS at the milk control station, was further studied. The dairy- and fieldmen of the two milk producers could not explain the reason for the bulk tank failures since no cows were treated during the preceding month nor had fresh cows been added to the milking herd. The purpose of the investigation was to establish whether antibiotic residues were present in the positive samples and to indicate the origin of inhibition by the silo milk. The results of the antimicrobial testing using the Delvotest MCS after addition of penicillinase or *p*-aminobenzoic acid to the milk, the Charm MRL Beta-lactam test and the Charm II assay, were confirming the opinion of the dairy- and fieldmen that the inhibition in the Delvotest was not caused by the presence of residues of antibiotics or chemotherapeutics.

In most of the positive milk samples a high lipolysis was found. This could be expected since a high SCC was found in the milk of 6 out of 11 cows. A high SCC could implicate an increased lipase activity, both endogenic and bacterial, leading to a higher concentration of short chain free fatty acids in this milk. An inhibitory activity by some fatty acids is described in literature. However, in our case there are several indications that the inhibition of the Delvotest was not caused by fat destruction and the release of fatty acids. Even when milk is spiked with high levels of fatty acids that occur most abundantly in milk (myristic, palmitic, stearic, and oleic acid) no inhibition of *Geobacillus* on the disc assay could be observed (data not shown), while large inhibition zones were obtained for the filter discs impregnated with milk after *Pseudomonas* growth, even after dialysis through a 1 kDa dialysis membrane.

From the VRBA plates with milk of cows 3 and 4, bacterial strains, surrounded by a clear halo were isolated, purified, and coded as P866 and P867. Both bacteria were identified as *Pseudomonas fluorescens* to 99.9% confidence level by API 20NE. *P. tolaasii* LMG 2342^T^ was indicated as the closest relative by phylogenetic clustering. The isolation of *Pseudomonas* strains is not surprising. The current practices for the collection and storage of the raw milk and the use of milking robots favor the growth of psychrotrophic bacteria like *Pseudomonas* spp., which is able to grow below 7°C [63], [58], [64], [31]. Significant contaminations by pseudomonads occur due to inadequately sanitized surfaces of milking, storage, and transporting equipment. Besides their rapid growth ability in refrigerated milk, psychrotrophs produce heat-stable extracellular proteases, lipases, and phospholipases. *Pseudomonas* spp. are the primary concern with regard to lipolytic degradation of milk fat [65]).

*Pseudomonas* spp. are also known to produce cyclic lipodepsipeptides. CLPs are composed of a fatty acid tail linked to a short oligopeptide, which is cyclised to form a lactone (depsi) ring between two amino acids in the peptide chain [66]. They are very diverse both structurally and in terms of their biological activity [67]. CLPs produced by *Pseudomonas* spp. play a key role in antimicrobial activity against a range of other micro-organisms [68], [69]. Activity against *Bacillus megaterium* was shown for corpeptins, syringopeptins, and tolaasin [70], [71]. Besides an antimicrobial activity, the CLPs cormycin A [72] and tolaasin showed erythrocyte haemolytic properties [59]. The extracellular CLP toxin, tolaasin, produced by *P. tolaasii*, is described as heat-stable [73], [74], [75].

Both strains, P866 and P867, are psychrophilic, hemolytic, and proteolytic. Inoculated milk incubated at 30°C or at 5–7°C showed fat oxidation and tested positively on the Delvotest. In comparison, *P. tolaasii* LMG 2342^T^ showed no haemolytic activity and a lower inhibitory effect. In all growth experiments, a high number of bacteria (>10^7^ ml^−1^) was needed before positive Delvotest results could be generated. These experimental findings are contradictory to the initial false-positive Delvotest MCS results at the milk control stations, generated by farm silo milk with a normal bacterial count. Literature [76] suggests that the presence of a bacterial biofilm could have an influence, or that quorum sensing is regulating the production of inhibitory substances. For example, the psychrotrophic bacterium *Pseudomonas fluorescens* uses quorum sensing to coordinate the formation of biofilms, exopolysaccharide production, and cell aggregation [77]. Perhaps the bacterial flora in the milk is inhibited by such production of antibacterial inhibitors, although the influence by a typical flora present at the farm cannot be excluded. Preliminary experiments with incubation of *Pseudomonas* inoculated in milk in the presence of other pseudomonads or the normal flora of blank raw milk showed no enhancement of bacterial inhibitor production but more likely the opposite. Possibly the *Pseudomonas* P867 strain does not produce toxins against other *Pseudomonas* strains.

The bacterial inhibitor by growth of *Pseudomonas* P867 in milk was not only inhibitory to *Geobacillus stearothermophilus* var. *calidolactis* but also to other Gram-positives (*B. cereus*, *B. subtilis, and Staphylococcus aureus*) while the Gram-negatives tested were not inhibited. Tolaasins also showed antimicrobial activity against Gram-positive bacteria. Bassarello and co-workers [78] assayed the antimicrobial activity of tolaasins A-E in comparison with tolaasin I and II against the Gram-positive bacteria *Bacillus megaterium* and *Rhodococcus fascians*, respectively, and the Gram-negative bacteria *Escherichia coli* and *Erwinia amylovora* subsp. *carotovora*. All the analogues, except tolaasin C, inhibited the growth of the tested Gram-positive bacteria, although differences among their specific activities were observed. However, none of the tested Gram-negative bacteria were inhibited. The minimal inhibitory quantity of tolaasin A to inhibit *Bacillus megaterium* was 1.28 µg [78].

The NMR analysis performed on the supernatant after centrifugation of a culture of Pseudomonas P867 identified the bacterial inhibitors as cyclic lipodepsipeptides. The four identified CLPs possess structures that clearly define them as members of the viscosin group [67] (Figure 2), a collection of CLPs with similar oligopeptide sequences produced by Pseudomonas spp. These compounds have been reported to display both antibiotic and antifungal activities [79], [80], [81], [82], likely by permeabilization of the cellular membrane [81], [83], [84], [85]. Until now, the observed variations in molecular constitution within the viscosin group have always been limited to residues at positions 2 (Glu or Gln), 4 (Ile, Val or Leu) and 9 (Ile, Val or Leu) or affected the fatty acid chain length (ranging from C_10_ to C_12_). The identified CLP structures **1–4** thus fall within the known molecular space sampled by the viscosin group. It should be noted that amongst these, only one variation exists in terms of molecular configuration, i.e. at position 5, which is either an L-Leu or D-Leu, effectively dividing the viscosin group into two subgroups. (Figure 2). Since no configurational information was obtained for the isolated CLPs, their exact identity and place within the viscosin group cannot be established with full certainty. Known viscosin group members with exactly the same constitution as **1** and **4** are either massetolides A and C [80] or pseudophomines A and B [86], [82]. A comparison of the ^1^H and ^13^C chemical shifts in acetone-d6 with those reported for the massetolides [80] clearly demonstrates a significant difference at the level of the Leu5 residue. (Figure S11, Tables S3 and S4 in File S1). On the other hand, comparing the ^1^H and ^13^C chemical shifts in DMF-d7 solution with those of WLIP [87], [88], a CLP from the D-Leu5-subgroup, reveals a much higher similarity for this residue. This provides a strong argument for attributing **1–4** to the D-Leu5-subgroup. (Figure S12 in File S1). It might therefore very well be that **1** and **4** correspond to pseudophomin A and B respectively, CLPs with reported antifungal activity produced by *Pseudomonas fluorescens* strain BRG100. Nevertheless, differences in configurations at other positions, most notably in the Ile4 and Ile9 side-chains cannot be excluded. Therefore, the precise identity of these CLPs cannot be established with full certainty.

### Final conclusions

To our knowledge this paper is the first statement of interference of microbial inhibitor tests for antibiotic residues in milk by cyclic lipodepsipeptides produced by *Pseudomonas* strains. The strains, isolated from milk of two farms with frequent problems of false-positive Delvotest results were identified as closely related to *Pseudomonas tolaasii*. Growth of the isolates in milk not only resulted in high lipolysis of the raw milk, but also in the production of heat-tolerant CLPs, shown to be members of the viscosin group. The CLPs are inhibitory to *Geobacillus stearothermophilus* var. *calidolactis*, the test strain used in most of the commercially available microbiological inhibitor tests. Interference in the Delvotest, Eclipse, Charm Blue Yellow II, and PremiTest was established, while the acid production in Copan Milk Test was also hampered, but not to the same extent, resulting in no false positive test results. The CLPs also showed antimicrobial activity against *Staphylococcus aureus*, *B. cereus*, and *B. subtilis*.

Our findings have a serious consequence for regulatory quality programmes. In such programmes, in most cases, a confirmation of initial positive screening results is foreseen, including a heat-treatment of the milk to exclude influence by natural inhibitors. However, the bacterial inhibitors produced by *Pseudomonas* P866 and P867 are heat-stable and will in most test schemes lead to false positive results. In some countries, special regulatory measures including higher financial penalties or a suspension of milk collection are imposed onto recidivists. Pseudomonads are known to be well adapted for survival in milk processing environment, as they are able to adhere strongly to the surface of milk processing equipment and are capable to colonize the milking equipment and storage tank at the farm. The production of bacterial inhibitors is very likely to be a persistent problem on certain farms. We speculate that they can occasionally produce enough inhibitors to cause positive inhibitor test results.

It remains difficult to give a rate of occurrence of this phenomenon. It is obvious, that in most cases positive inhibitor tests are caused by the presence of residues of antibiotics, mainly belonging to the family of β-lactams. Nevertheless, our data show that results of microbiological inhibitor tests should be interpreted with care, especially when the outcome of such tests is not confirmed by a confirmatory method, providing full or complementary information enabling the substance to be unequivocally identified.

Our findings indicate a new challenge for the dairy industry. By extending the refrigerated storage of milk, the keeping quality of milk is influenced by growth and metabolic activities of psychrotrophic bacteria at low temperatures. This not only results in possible spoilage of long-life milk and the production of yoghurt of inferior quality, but also in false-positive microbial inhibitor tests.

## Supporting Information

File S1 **File includes Figures S1–S12 and Tables S1–S4.** Figure S1: LC-MS chromatogram of the acidified mixture prior to purification using UV-detection at 214 nm. The isolated fractions are indicated with braces and the individual peptides with arrows. Figure S2: Mass spectra of isolated lipopeptides 1–4 obtained during LC-MS analysis of the extracted mixture prior to purification. The range of detectable m/z values was limited to 1500 Da, ESI (Positive mode). Figure S3: 1D ^1^H spectrum of peptide 4 in DMF-d7 solution, 25°C, 700 MHz. Figure S4: 2D ^1^H-^1^H TOCSY spectrum of peptide 4 in acetone-d6 solution, 25°C, 500 MHz, revealing the characteristic amino acid spin system patterns. Figure S5: Established ^1^H-^1^H ROE contacts in peptide 4, observed in a 2D ^1^H-^1^H ROESY spectrum with 300 ms mixing time in DMF-d7 solution. Figure S6: ^3^JCH contacts between Thr3 H^β^ and carbonyl carbons of peptide 4, observed in a 2D ^1^H–^13^C HMBC experiment, 25°C, 700 MHz, in DMF-d7 solution. Figure S7: High resolution mass spectrum of peptide 4. Figure S8: 1D ^1^H spectrum of peptide 1 in DMF-d7 solution, 25°C, 700 MHz. A) H^N^ region, B) H^α^ region and C) aliphatic region spectrum. Figure S9: High resolution mass spectrum of peptide 1. Figure S10: High resolution mass spectrum of peptides 2/3. Figure S11: A diagram plotting the ^1^H and ^13^C chemical shifts of the CH^α^ units of massetolide A (blue) from Gerard et al, J. Nat. Prod. (1997) 60:223–229, and peptide 1 (red) in acetone-d6 solution recorded at 500 MHz, 25°C. The arrow indicates the remarkable difference in chemical shift of the 5^th^ residue, suggesting a difference in configuration. Figure S12: Overlay of 2D ^1^H-^13^C HSQC spectra of peptide 1 (blue) and WLIP (red) in DMF-d7 solution, recorded at 500 MHz, 25°C, CHα-region. The similarity for the chemical shift of the Leu5 residue suggests the milk peptides 1–4 belong to the D-subgroup of CLPs. Table S1: ^1^H and ^13^C NMR assignment of peptide 4 in DMF-d7 solution, 25°C, 700 MHz. Table S2: ^1^H and ^13^C NMR assignment of peptide 1 in DMF-d7 solution, 25°C, 700 MHz. Table S3: ^1^H and ^13^C NMR assignment of peptide 1 in acetone-d6 solution, 25°C, 500 MHz. Table S4: ^1^H and ^13^C NMR assignment of peptide 4 in acetone-d6 solution, 25°C, 500 MHz.
(DOCX)
