# Supplementary material for: Cyclic Lipodepsipeptides Produced by Pseudomonas spp. Naturally Present in Raw Milk Induce Inhibitory Effects on Microbiological Inhibitor Assays for Antibiotic Residue Screening
Source: PLoS One. 2014 May 22;9(5):e98266. doi: 10.1371/journal.pone.0098266 (PMC4031126; doi:10.1371/journal.pone.0098266)
Supplement: File S1 — File includes Figures S1–S12 and Tables S1–S4. Figure S1: LC-MS chromatogram of the acidified mixture prior to purification using UV-detection at 214 nm. The isolated fractions are indicated with braces and the individual peptides with arrows. Figure S2: Mass spectra of isolated lipopeptides 1–4 obtained during LC-MS analysis of the extracted mixture prior to purification. The range of detectable m/z values was limited to 1500 Da, ESI (Positive mode). Figure S3: 1D 1H spectrum of peptide 4 in DMF-d7 solution, 25°C, 700 MHz. Figure S4: 2D 1H-1H TOCSY spectrum of peptide 4 in acetone-d6 solution, 25°C, 500 MHz, revealing the characteristic amino acid spin system patterns. Figure S5: Established 1H-1H ROE contacts in peptide 4, observed in a 2D 1H-1H ROESY spectrum with 300 ms mixing time in DMF-d7 solution. Figure S6: 3JCH contacts between Thr3 Hβ and carbonyl carbons of peptide 4, observed in a 2D 1H–13C HMBC experiment, 25°C, 700 MHz, in DMF-d7 solution. Figure S7: High resolution mass spectrum of peptide 4. Figure S8: 1D 1H spectrum of peptide 1 in DMF-d7 solution, 25°C, 700 MHz. A) HN region, B) Hα region and C) aliphatic region spectrum. Figure S9: High resolution mass spectrum of peptide 1. Figure S10: High resolution mass spectrum of peptides 2/3. Figure S11: A diagram plotting the 1H and 13C chemical shifts of the CHα units of massetolide A (blue) from Gerard et al, J. Nat. Prod. (1997) 60:223–229, and peptide 1 (red) in acetone-d6 solution recorded at 500 MHz, 25°C. The arrow indicates the remarkable difference in chemical shift of the 5th residue, suggesting a difference in configuration. Figure S12: Overlay of 2D 1H-13C HSQC spectra of peptide 1 (blue) and WLIP (red) in DMF-d7 solution, recorded at 500 MHz, 25°C, CHα-region. The similarity for the chemical shift of the Leu5 residue suggests the milk peptides 1–4 belong to the D-subgroup of CLPs. Table S1: 1H and 13C NMR assignment of peptide 4 in DMF-d7 solution, 25°C, 700 MHz. Table S2: 1H and 13C NMR assi [file pone.0098266.s001.docx]

**File S1. Supporting information**

**Figure S1.** **LC-MS chromatogram of the acidified mixture prior to purification using UV-detection at 214 nm.** The isolated fractions are indicated with braces and the individual peptides with arrows.

| Retention time | Fraction | m/z ^a^ | Quantity ^b^ |
| --- | --- | --- | --- |
| 6.5 min. | A | 1140.7 | 3.3 mg |
| 8.1 and 8.3 min. | B | 1154.7 ^c^ | 3.6 mg |
| 9.2 min. | C | 1168.7 | 5.7 mg |

^a^ positive mode

^b^ total amount of isolated peptide

^c^ masses of peptides 2 and 3 are identical

**Figure S2. Mass spectra of isolated lipopeptides 1-4 obtained during LC-MS analysis of the extracted mixture prior to purification.** The range of detectable m/z values was limited to 1500 Da, ESI (Positive mode).

impurity

DMF HCO

**Figure S3. 1D ^1^H spectrum of peptide 4 in DMF-d7 solution, 25°C, 700 MHz.**

A) H^N^ region, B) H^α^ region and C) aliphatic region spectrum.

**Table S1. ^1^H and ^13^C NMR assignment of peptide 4 in DMF-d7 solution, 25°C, 700MHz.**

**Table S1. ^1^H and ^13^C NMR assignment of peptide 4 in DMF-d7 solution, 25°C, 700MHz (continued).**

**Figure S4.** **2D ^1^H-^1^H TOCSY spectrum of peptide 4 in acetone-d6 solution, 25°C, 500 MHz, revealing the characteristic amino acid spin system patterns.**

**Figure S5.** **Established ^1^H-^1^H ROE contacts in peptide 4, observed in a 2D ^1^H-^1^H ROESY spectrum with 300 ms mixing time in DMF-d7 solution**.


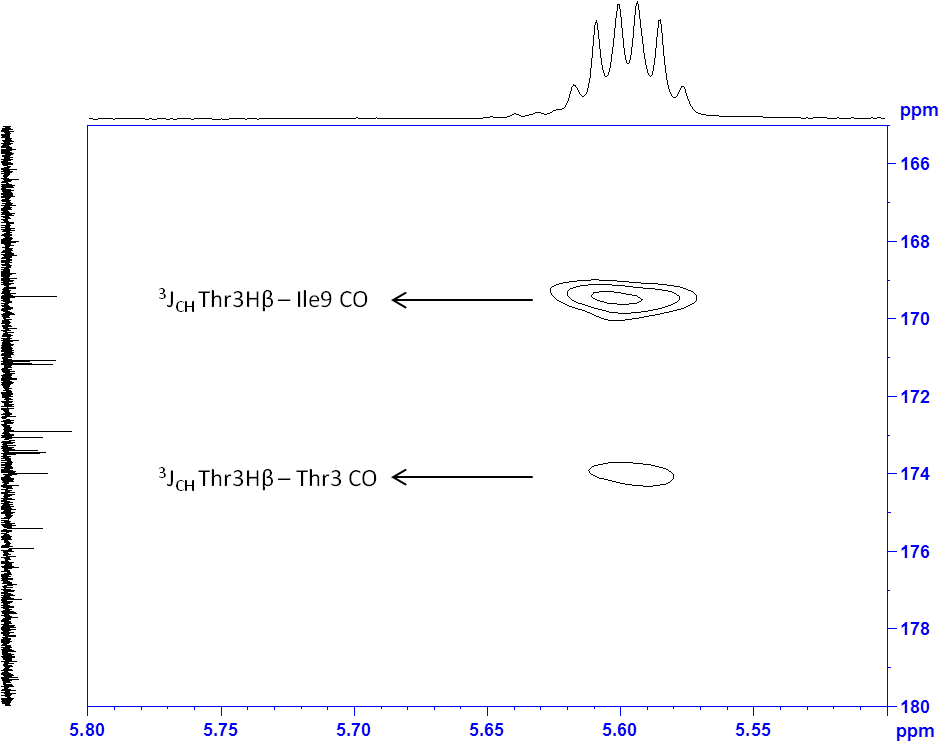


**Figure S6.** **^3^J_CH_ contacts between Thr3 H^β^ and carbonyl carbons of peptide 4, observed in a 2D ^1^H-^13^C HMBC experiment, 25°C, 700 MHz, in DMF-d7 solution**.

**A**


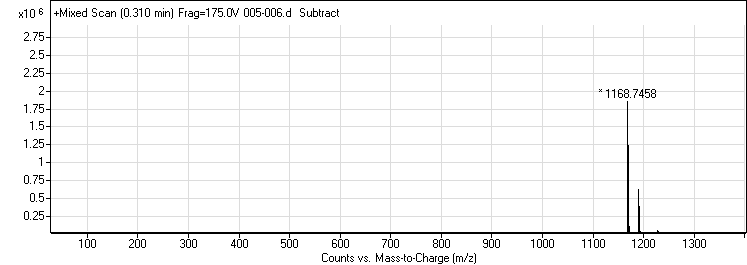


**B**


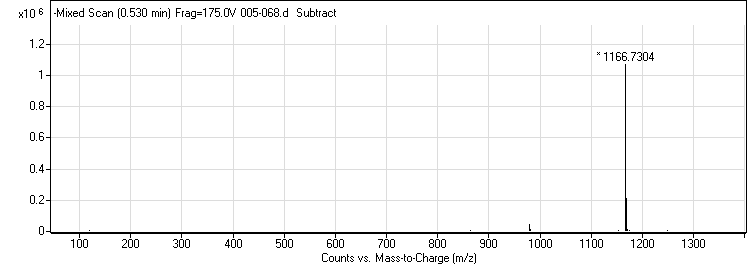


**Figure S7. High resolution mass spectrum of peptide 4.**

A: full mass spectrum (positive mode)

B: full mass spectrum (negative mode)

Expected exact mass of peptide 4 (C_56_H_99_N_9_O_16_) + H^+^: 1168.7439 Da

Observed exact mass of peptide 4 + H^+^: 1168.7458 Da

$$\Delta=\frac{1168.7458-1168.7439}{1168.7439}\times{10}^{6}\mathrm{ppm}=1.6 \mathrm{ppm}$$

Expected exact mass of peptide 4 (C_57_H_101_N_9_O_16_) - H^+^: 1166.7293 Da

Observed exact mass of peptide 4 - H^+^: 1166.7304 Da

$$\Delta=\frac{1166.7304-1166.7293}{1166.7293}\times{10}^{6}\mathrm{ppm}=0.9 \mathrm{ppm}$$


**A**

DMF HCO

**B**

**C**

.

**Figure S8.** **1D ^1^H spectrum of peptide 1 in DMF-d7 solution, 25°C, 700 MHz.**

A) H^N^ region, B) H^α^ region and C) aliphatic region spectrum.

**A**


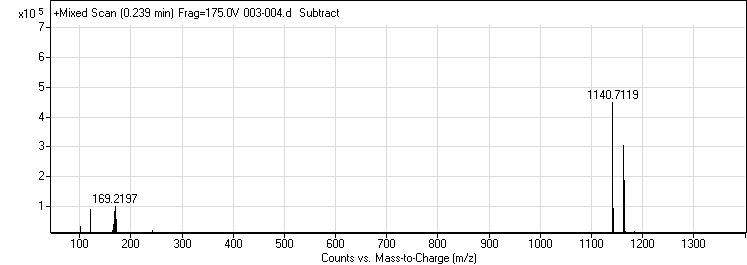


**B**


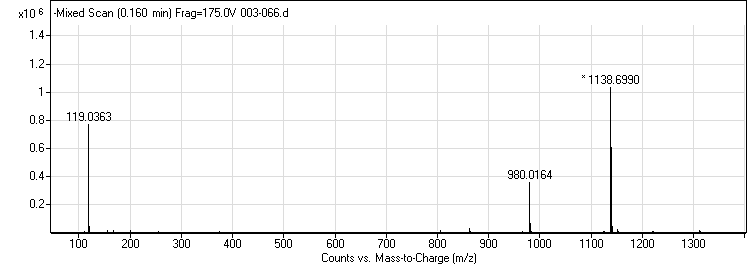


**Figure S9. High resolution mass spectrum of peptide 1.**

A: full mass spectrum (positive mode)

B: full mass spectrum (negative mode)

Expected exact mass of peptide 1 (C_55_H_97_N_9_O_16_)+ H^+^: 1140.7126 Da

Observed exact mass of peptide 1 + H^+^: 1140.7119 Da

$$\Delta=\frac{1140.7119-1140.7126}{1140.7126}\times{10}^{6}\mathrm{ppm}=-0.6 \mathrm{ppm}$$

Expected exact mass of peptide 1 (C_55_H_97_N_9_O_16_) - H^+^: 1138.6981 Da

Observed exact mass of peptide 1 − H^+^: 1138.6990 Da

$$\Delta=\frac{1138.6990-1138.6981}{1138.6981}\times{10}^{6}\mathrm{ppm}=0.8 \mathrm{ppm}$$

**Table S2. ^1^H and ^13^C NMR assignment of peptide 1 in DMF-d7 solution, 25°C, 700MHz.**

**Table S2. ^1^H and ^13^C NMR assignment of peptide 1 in DMF-d7 solution, 25°C, 700MHz (continued).**

**A**


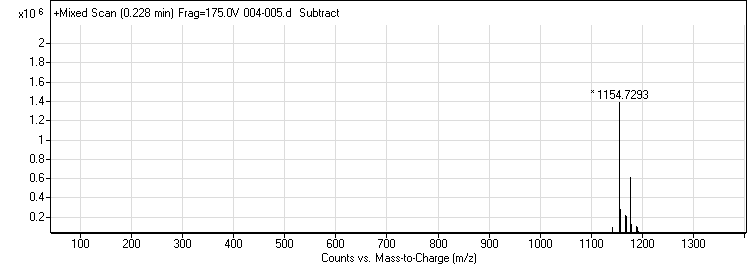


**B**


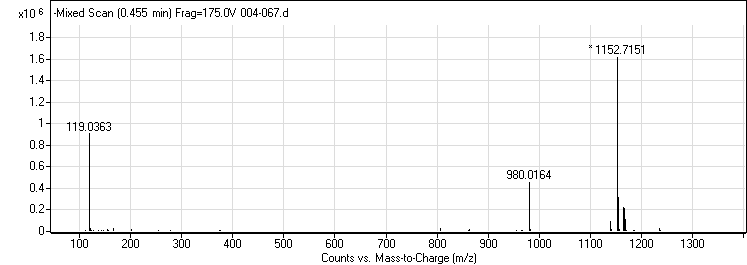


**Figure S10. High resolution mass spectrum of peptides 2/3.**

A: full mass spectrum (positive mode)

B: full mass spectrum (negative mode)

Expected exact mass of peptide 2/3 (C_56_H_99_N_9_O_16_) + H^+^: 1154.7283 Da

Observed exact mass of peptide 2/3 + H^+^: 1154.7293 Da

$$\Delta=\frac{1154.7293-1154.7283}{1154.7283}\times{10}^{6}\mathrm{ppm}=0.9 \mathrm{ppm}$$

Expected exact mass of peptide 2/3 (C_55_H_97_N_9_O_16_) - H^+^: 1152.7137 Da

Observed exact mass of peptide 2/3 − H^+^: 1152.7151 Da

$$\Delta=\frac{1152.7151-1152.7137}{1152.7137}\times{10}^{6}\mathrm{ppm}=1.2 \mathrm{ppm}$$


**Figure S11. A diagram plotting the ^1^H and ^13^C chemical shifts of the CH^α^ units of massetolide A (blue) from Gerard *et al*, J. Nat. Prod. (1997) 60:223-229, and peptide 1 (red) in acetone-d6 solution recorded at 500 MHz, 25°C.** The arrow indicates the remarkable difference in chemical shift of the 5^th^ residue, suggesting a difference in configuration.

**Table S3. ^1^H and ^13^C NMR assignment of peptide 1 in acetone-d6 solution, 25°C, 500MHz.**

|  |  |  | ^1^H δ [ppm] | ^13^C δ [ppm] |  |  |  | ^1^H δ [ppm] | ^13^C δ [ppm] |
| --- | --- | --- | --- | --- | --- | --- | --- | --- | --- |
| **HDA** |  | CO |  | n.a. | **allo-Thr3** |  |  |  |  |
|  |  | CH_2_ α1 | 2.56 | 44.37 | ^3^J_HNHα_ [Hz] | n.d. | NH | 8.39 |  |
|  |  | CH_2_ α2 | 2.56 | 44.37 | ^3^J_HαHβ_ [Hz] | n.d. | CH α | 4.14 | 61.45 |
|  |  | CH β | 4.11 | 69.41 |  |  | CO |  | n.a. |
|  |  | CH_2_ γ | 1.59 | 38.20 |  |  | CH β | 5.42 | 69.83 |
|  |  | CH_2_ δ1 | 1.38 | 26.27 |  |  | CH_3_ γ | 1.37 | 18.38 |
|  |  | CH_2_ δ2 | 1.50 | 26.27 |  |  |  |  |  |
|  |  | CH_2_ ε | 1.30 | 29.96 | **Ile4** |  |  |  |  |
|  |  | CH_2_ ζ | 1.30 | 29.96 | ^3^J_HNHα_ [Hz] | n.d. | NH | 7.72 |  |
|  |  | CH_2_ η | 1.28 | 32.35 |  |  | CH α | 3.68 | 63.76 |
|  |  | CH_2_ θ | 1.29 | 23.08 |  |  | CO |  | n.a. |
|  |  | CH_3_ ι | 0.88 | 14.17 |  |  | CH β | 2.15 | 35.45 |
|  |  | OH | n.a. |  |  |  | CH_3_ γ | 1.04 | 16.89 |
|  |  |  |  |  |  |  | CH_2_ γ1 | 1.19 | 26.05 |
|  |  |  |  |  |  |  | CH_2_ γ2 | 1.53 | 26.05 |
| **Leu1** |  |  |  |  |  |  | CH_3_ δ | 0.93 | 10.45 |
| ^3^J_HNHα_ [Hz] | n.d. | NH | 8.84 |  |  |  |  |  |  |
|  |  | CH α | 4.08 | 53.18 | **Leu5** |  |  |  |  |
|  |  | CO |  | n.a. | ^3^J_HNHα_ [Hz] | n.d. | NH | 7.89 |  |
|  |  | CH_2_ β1 | 1.85 | 39.00 |  |  | CH α | 4.06 | 55.12 |
|  |  | CH_2_ β2 | 1.85 | 39.00 |  |  | CO |  | n.a. |
|  |  | CH γ | 1.76 | 25.23 |  |  | CH_2_ β1 | 1.54 | 40.24 |
|  |  | CH_3_ δ | 0.93 | 21.87 |  |  | CH_2_ β2 | 1.76 | 40.24 |
|  |  | CH_3_ δ | 0.97 | 23.42 |  |  | CH γ | 1.89 | 25.26 |
| **Glu2** |  |  |  |  |  |  | CH_3_ δ | 0.88 | 20.98 |
| ^3^J_HNHα_ [Hz] | n.d. | NH | 8.79 |  |  |  | CH_3_ δ | 0.88 | 23.23 |
|  |  | CH α | 4.14 | 57.06 |  |  |  |  |  |
|  |  | CO |  | n.a. |  |  |  |  |  |
|  |  | CH_2_ β | 2.09 | 26.42 |  |  |  |  |  |
|  |  | CH_2_ γ | 2.53 | 30.73 |  |  |  |  |  |
|  |  | COOH δ |  | n.a. |  |  |  |  |  |
|  |  |  |  |  |  |  |  |  |  |

n.d. = not determined

n.a. = not assigned

**Table S3. ^1^H and ^13^C NMR assignment of peptide 1 in acetone-d6 solution, 25°C, 500MHz (continued).**

|  |  |  | ^1^H δ [ppm] | ^13^C δ [ppm] |  |  |  | ^1^H δ [ppm] | ^13^C δ [ppm] |
| --- | --- | --- | --- | --- | --- | --- | --- | --- | --- |
| **Ser6** |  |  |  |  | **Ser8** |  |  |  |  |
| ^3^J_HNHα_ [Hz] | 6.05 | NH | 7.20 |  | ^3^J_HNHα_ [Hz] | 8.88 | NH | 8.01 |  |
|  |  | CH α | 4.40 | 56.02 |  |  | CH α | 4.51 | 56.51 |
|  |  | CO |  | n.a. |  |  | CO |  | n.a. |
|  |  | CH_2_ β1 | 3.85 | 64.10 |  |  | CH_2_ β1 | 3.67 | 62.83 |
|  |  | CH_2_ β2 | 4.18 | 64.12 |  |  | CH_2_ β2 | 3.93 | 62.80 |
|  |  | OH γ | n.a. |  |  |  | OH γ | n.a. |  |
| **Leu7** |  |  |  |  | **Ile9** |  |  |  |  |
| ^3^J_HNHα_ [Hz] | 5.23 | NH | 7.24 |  | ^3^J_HNHα_ [Hz] | 9.86 | NH | 6.69 |  |
|  |  | CH α | 4.21 | 54.56 |  |  | CH α | 4.58 | 56.82 |
|  |  | CO |  | n.a. |  |  | CO |  | n.a. |
|  |  | CH_2_ β1 | 1.64 | 41.64 |  |  | CH β | 2.01 | 36.70 |
|  |  | CH_2_ β2 | 1.97 | 41.64 |  |  | CH_3_ γ | 0.87 | 15.95 |
|  |  | CH γ | 2.02 | 25.04 |  |  | CH_2_ γ1 | 1.06 | 24.92 |
|  |  | CH_3_ δ | 0.91 | 21.32 |  |  | CH_2_ γ2 | 1.27 | 24.92 |
|  |  | CH_3_ δ | 1.02 | 23.35 |  |  | CH_3_ δ | 0.91 | 12.07 |

n.d. = not determined

n.a. = not assigned

**Table S4. ^1^H and ^13^C NMR assignment of peptide 4 in acetone-d6 solution, 25°C, 500MHz.**

|  |  |  | ^1^H δ [ppm] | ^13^C δ [ppm] |  |  |  | ^1^H δ [ppm] | ^13^C δ [ppm] |
| --- | --- | --- | --- | --- | --- | --- | --- | --- | --- |
| **HDA** |  | CO |  | 173.76 | **allo-Thr3** |  |  |  |  |
|  |  | CH_2_ α1 | 2.56 | 44.37 | ^3^J_HNHα_ [Hz] | 8.95 | NH | 8.03 |  |
|  |  | CH_2_ α2 | 2.56 | 44.37 | ^3^J_HαHβ_ [Hz] | n.d. | CH α | 4.14 | 61.45 |
|  |  | CH β | 4.11 | 69.41 |  |  | CO |  | n.a. |
|  |  | CH_2_ γ | 1.59 | 38.20 |  |  | CH β | 5.42 | 69.83 |
|  |  | CH_2_ δ1 | 1.38 | 26.27 |  |  | CH_3_ γ | 1.37 | 18.38 |
|  |  | CH_2_ δ2 | 1.50 | 26.27 |  |  |  |  |  |
|  |  | CH_2_ ε | 1.30 | 29.96 | **Ile4** |  |  |  |  |
|  |  | CH_2_ ζ | 1.30 | 29.96 | ^3^J_HNHα_ [Hz] | 5.54 | NH | 7.74 |  |
|  |  | CH_2_ η | 1.30 | 29.96 |  |  | CH α | 3.68 | 63.76 |
|  |  | CH_2_ θ | 1.30 | 29.96 |  |  | CO |  | 173.50 |
|  |  | CH_2_ ι | 1.28 | 32.35 |  |  | CH β | 2.15 | 35.45 |
|  |  | CH_2_ κ | 1.29 | 23.08 |  |  | CH_3_ γ | 1.04 | 16.89 |
|  |  | CH_3_ λ | 0.88 | 14.17 |  |  | CH_2_ γ1 | 1.19 | 26.05 |
|  |  | OH | n.a. |  |  |  | CH_2_ γ2 | 1.53 | 26.05 |
| **Leu1** |  |  |  |  |  |  | CH_3_ δ | 0.93 | 10.45 |
| ^3^J_HNHα_ [Hz] | 5.00 | NH | 8.95 |  |  |  |  |  |  |
|  |  | CH α | 4.04 | 53.24 | **Leu5** |  |  |  |  |
|  |  | CO |  | n.a. | ^3^J_HNHα_ [Hz] | 3.15 | NH | 7.92 |  |
|  |  | CH_2_ β1 | 1.85 | 39.00 |  |  | CH α | 4.06 | 55.12 |
|  |  | CH_2_ β2 | 1.85 | 39.00 |  |  | CO |  | 171.99 |
|  |  | CH γ | 1.76 | 25.23 |  |  | CH_2_ β1 | 1.54 | 40.24 |
|  |  | CH_3_ δ | 0.93 | 21.87 |  |  | CH_2_ β2 | 1.76 | 40.24 |
|  |  | CH_3_ δ | 0.97 | 23.42 |  |  | CH γ | 1.89 | 25.26 |
| **Glu2** |  |  |  |  |  |  | CH_3_ δ | 0.88 | 20.98 |
| ^3^J_HNHα_ [Hz] | n.d. | NH | 9.28 |  |  |  | CH_3_ δ | 0.88 | 23.23 |
|  |  | CH α | 4.11 | 57.45 |  |  |  |  |  |
|  |  | CO |  | n.a. |  |  |  |  |  |
|  |  | CH_2_ β | 2.09 | 26.42 |  |  |  |  |  |
|  |  | CH_2_ γ | 2.49 | 32.12 |  |  |  |  |  |
|  |  | COOH δ | n.a. | n.a. |  |  |  |  |  |
|  |  |  |  |  |  |  |  |  |  |

n.d. = not determined

n.a. = not assigned

**Table S4. ^1^H and ^13^C NMR assignment of peptide 4 in acetone-d6 solution, 25°C, 500MHz (continued).**

|  |  |  | ^1^H δ [ppm] | ^13^C δ [ppm] |  |  |  | ^1^H δ [ppm] | ^13^C δ [ppm] |
| --- | --- | --- | --- | --- | --- | --- | --- | --- | --- |
| **Ser6** |  |  |  |  | **Ser8** |  |  |  |  |
| ^3^J_HNHα_ [Hz] | 8.62 | NH | 7.21 |  | ^3^J_HNHα_ [Hz] | 8.91 | NH | 8.03 |  |
|  |  | CH α | 4.40 | 56.02 |  |  | CH α | 4.51 | 56.51 |
|  |  | CO |  | 170.78 |  |  | CO |  | 170.83 |
|  |  | CH_2_ β1 | 3.85 | 64.10 |  |  | CH_2_ β1 | 3.67 | 62.83 |
|  |  | CH_2_ β2 | 4.18 | 64.12 |  |  | CH_2_ β2 | 3.93 | 62.80 |
|  |  | OH γ | n.a. |  |  |  | OH γ | n.a. |  |
| **Leu7** |  |  |  |  | **Ile9** |  |  |  |  |
| ^3^J_HNHα_ [Hz] | 6.16 | NH | 7.25 |  | ^3^J_HNHα_ [Hz] | 10.01 | NH | 6.71 |  |
|  |  | CH α | 4.21 | 54.56 |  |  | CH α | 4.58 | 56.82 |
|  |  | CO |  | 172.55 |  |  | CO |  | 168.93 |
|  |  | CH_2_ β1 | 1.64 | 41.64 |  |  | CH β | 2.01 | 36.70 |
|  |  | CH_2_ β2 | 1.97 | 41.64 |  |  | CH_3_ γ | 0.87 | 15.95 |
|  |  | CH γ | 2.02 | 25.04 |  |  | CH_2_ γ1 | 1.06 | 24.92 |
|  |  | CH_3_ δ | 0.91 | 21.32 |  |  | CH_2_ γ2 | 1.27 | 24.92 |
|  |  | CH_3_ δ | 1.02 | 23.35 |  |  | CH_3_ δ | 0.91 | 12.07 |

n.d. = not determined

n.a. = not assigned

**
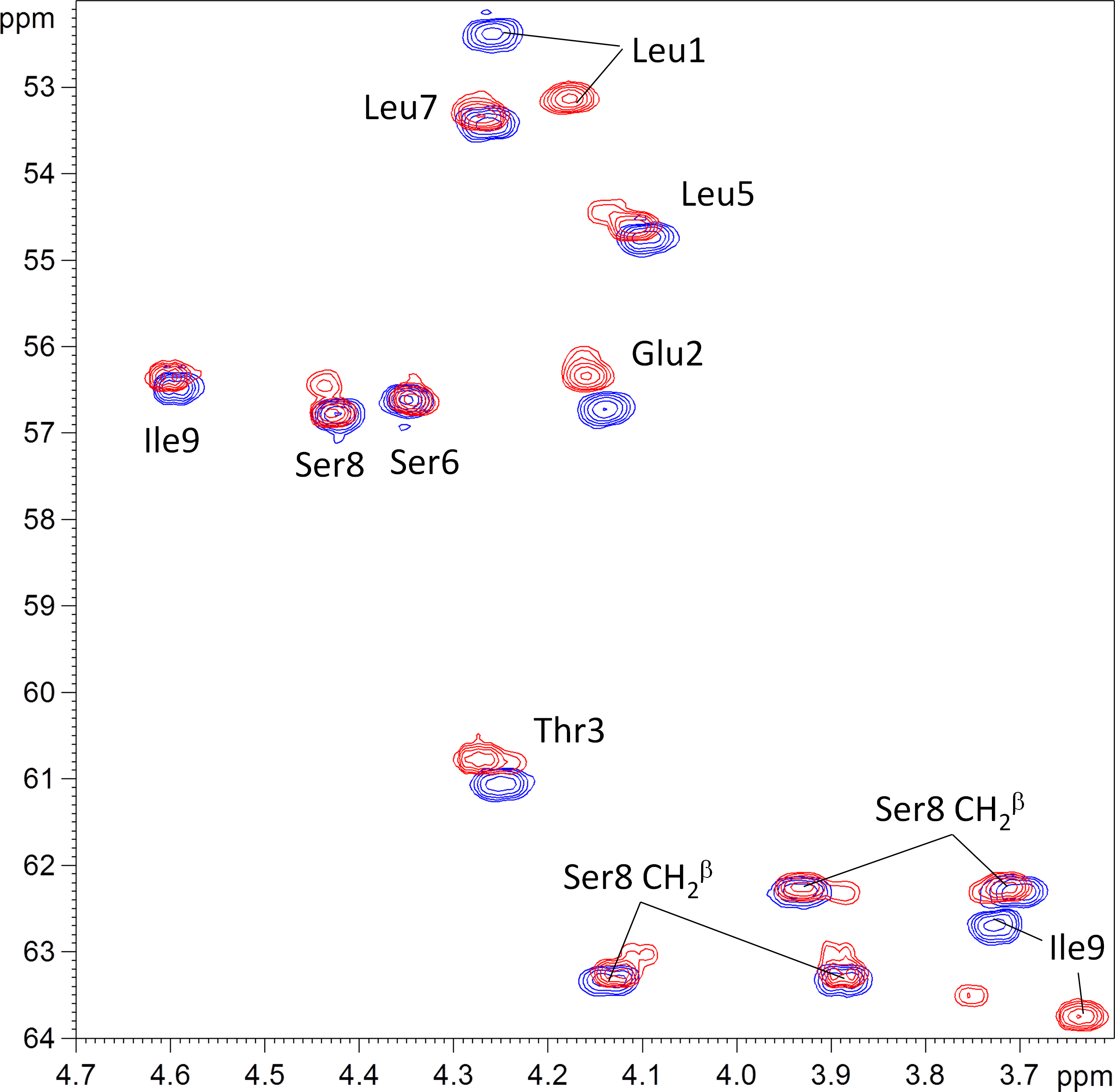
**

**Figure S12.** **Overlay of 2D ^1^H-^13^C HSQC spectra of peptide 1 (blue) and WLIP (red) in DMF-d7 solution, recorded at 500 MHz, 25°C, CHα-region.** The similarity for the chemical shift of the Leu5 residue suggests the milk peptides **1**-**4** belong to the D-subgroup of CLPs.
